# Reduced Photoinhibition under Low Irradiance Enhanced Kacip Fatimah (*Labisia pumila* Benth) Secondary Metabolites, Phenyl Alanine Lyase and Antioxidant Activity

**DOI:** 10.3390/ijms13055290

**Published:** 2012-04-25

**Authors:** Mohd Hafiz Ibrahim, Hawa Z.E. Jaafar

**Affiliations:** Department of Crop Science, Faculty of Agriculture, University Putra Malaysia, 43400 Serdang, Selangor, Malaysia; E-Mail: mhafizphd@yahoo.com

**Keywords:** carbon-based secondary metabolites, maximum efficiency of photosystem II, net photosynthesis, polyphenolics, PAL activity, DPPH assay

## Abstract

A randomized complete block design experiment was designed to characterize the relationship between production of total flavonoids and phenolics, anthocyanin, photosynthesis, maximum efficiency of photosystem II (Fv/Fm), electron transfer rate (Fm/Fo), phenyl alanine lyase activity (PAL) and antioxidant (DPPH) in *Labisia pumila* var. *alata*, under four levels of irradiance (225, 500, 625 and 900 μmol/m^2^/s) for 16 weeks. As irradiance levels increased from 225 to 900 μmol/m^2^/s, the production of plant secondary metabolites (total flavonoids, phenolics and antocyanin) was found to decrease steadily. Production of total flavonoids and phenolics reached their peaks under 225 followed by 500, 625 and 900 μmol/m^2^/s irradiances. Significant positive correlation of production of total phenolics, flavonoids and antocyanin content with Fv/Fm, Fm/Fo and photosynthesis indicated up-regulation of carbon-based secondary metabolites (CBSM) under reduced photoinhibition on the under low light levels condition. At the lowest irradiance levels, *Labisia pumila* extracts also exhibited a significantly higher antioxidant activity (DPPH) than under high irradiance. The improved antioxidative activity under low light levels might be due to high availability of total flavonoids, phenolics and anthocyanin content in the plant extract. It was also found that an increase in the production of CBSM was due to high PAL activity under low light, probably signifying more availability of phenylalanine (Phe) under this condition.

## 1. Introduction

Phenolics are carbon-based secondary metabolites, primarily produced through the pentose phosphate pathway (PPP), phenylpropanoid and shikimate acid pathways. The oxidative PPP provides precursor erythrose-4-phosphate for the shikimate pathway. This pathway converts these sugar phosphates to aromatic amino acids such as phenylalanine, which becomes the precursor for the phenylpropanoid pathway that synthesizes polyphenols. In plant cells, simple polyphenolics are believed to be scavengers of free radicals, protecting the cells from free radical damage. Phenolics are also involved in the strengthening of plant cell walls during growth by polymerization into lignans and lignins [1,2]. Plant phenolics have potential health benefits, mainly due to their antioxidant properties, such as reactive oxygen species scavenging and inhibition, electrophilic scavenging, and metal chelation [3]. Epidemiological studies support a relationship between the consumption of phenolic-rich food products and a low incidence of coronary heart disease, atherosclerosis, certain forms of cancer and stroke [4–7]. Plant phenolics have also been reported to exhibit pharmacological properties such as antitumor, antiviral, antimicrobial, anti-inflammatory, hypotensive and antioxidant activity [8,9].

The concentration of polyphenols has been found to be influenced by environmental conditions, which can change the concentration of these active constituents [10]. Irradiance (photosynthetic photon flux density, PPFD; 400–700 nm) is known to regulate not only plant growth and development, but also the biosynthesis of both primary and secondary metabolites [11,12]. The carbon nutrient balance (CNB) hypothesis predicts that the pattern of allocation to carbon-based secondary metabolites (CBSM) depends on the relative availability of carbon and nutrients, as well as on their relationship with plant growth rate. In accord with the predictions, the stimulation of secondary metabolite production has been demonstrated under high light [13] or low light; [14] and by other biotic and abiotic stresses, e.g., pathogen attack, mechanical wounding, ultraviolet (UV) radiation, low temperatures and nutrient deficit [15–17]. Phenylalanine ammonia-lyase enzyme (PAL) catalyzes the conversion of phenylalanine into trans-cinnamic acid, thereby causing the flux of primary metabolites into secondary metabolites in the phenolics pathway. PAL activity and other phenylpropanoid enzymes are mainly regulated via *de novo* synthesis. Hemm *et al.* [13] showed that PAL genes were highly expressed in the low light-grown Arabidopsis roots, and Fritz *et al.* [18] reported that PAL gene expression could be induced by nitrate deficiency in *Nicotiana tabacum* plants. In fact, low light intensities were regarded as environmental stimuli for the production of secondary metabolites in some plant species [19–21]. Previous studies report an increase in the concentration of specific secondary metabolites in some medicinal species, such as methylxanthines in *Ilex paraguariensis* [19], alkaloid concentration in *Delphinium barbeyi* [20], and aloin (barbaloin) in *Aloe mutabilis* [22,23], under low light conditions. There were also numerous reports on an increase of secondary metabolites under high light condition and has been reported in tea [24], red kidney bean [25] and St. John’s wort [26]. These showed the importance of light in secondary metabolite regulation in medicinal plants.

In general, plants living in an exposed full sun habitat exhibit different photosynthetic properties and leaf characteristics than those living in the shade. Tropical pioneer species show higher responsiveness to high irradiance than late-successional species [27]. However, under high irradiance, plants are predisposed to suffer photoinhibition, which is defined as the slow, reversible decline in photosynthetic efficiency that occurs when the absorbed light is in excess of that required for carbon assimilation [28,29]. This phenomenon is very frequent in tropical plants [30], where the light intensity can reach levels beyond 1800 μmol/m^2^/s photosynthetic photon flux density (PPFD) [31]. The ability to cope with photoinhibition varies between different plant species [32,33]. Photoinhibition occurs because shade leaves have high concentrations of chlorophyll, thus a higher light-capturing capacity, due to the larger antenna size of the photosystem II (PSII), and lower rates of light-saturated photosynthesis due to lower amounts of photosynthetic enzymes, such as Rubisco. A previous study by Close *et al.* [34] has shown that photoinhibition can enhanced the production of polyphenolics in *Eucalytus* plants. The result implies that production of plant secondary metabolites can be enhanced by the manipulation of irradiance.

*Labisia pumila* Benth., locally known as Kacip Fatimah, Selusoh Fatimah or Akar Kacip Fatimah, is a sub-herbaceous plant with creeping stems from the family Myrsinaceae that is found widespread in Indochina and throughout Malaysian forests [35]. Stone [36] has categorized three varieties of this herb in Malaysia, namely *Labisia pumila* var. *alata*, *Labisia pumila* var. *pumila* and *Labisia pumila* var. *lanceolata*. Each of these varieties has different uses. These uses include treatments for dysentery, dysmenorrhea, flatulence and gonorrhea [37]. Previous studies have indicated that the bioactive compounds of *Labisia pumila* consist of resorcinols, flavonoids and phenolic acids [38–42]. These compounds are phenolics, and possess a wide range of structures that contribute to the organoleptic and nutritional qualities of fruits and vegetables and can be enriched by micro-climatic manipulation [43–45]. There have been numerous reports on the chemical profiling and antioxidative effects of the plant [43,44], however, there were less studies undertaken on microenvironmental manipulation, especially the effects of irradiance on the secondary metabolites and antioxidant activity of *Labisia pumila*. A previous study by Jaafar *et al.* [35] has shown that the manipulation of irradiance (photosynthetic photon flux density) was able to enhance the production of secondary metabolites in this plant; however, there are no results on photosynthetic performance, photosystem II acclimation and antioxidant activity of the plant under different irradiances levels. So, the objective of the study was to examine the effects of different irradiance levels on maximal quantum efficiency of photosystem II (Fv/Fm), electron transfer rate (Fm/Fo), photosynthetic rates, PAL activity, secondary metabolite and antioxidant activities in *Labisia pumila*. Relationships among the parameters were also determined.

## 2. Results and Discussion

### 2.1. Total Flavonoids and Phenolics Profiling

Irradiance levels had a significant (*P* ≤ 0.05) impact on the production of total flavonoids and phenolics (Table 1). As irradiance levels decreased from 900 to 225 μmol/m^2^/s, *Labisia pumila* produced more total flavonoids and phenolics. It was found that the accumulation pattern of secondary metabolites was more pronounced in leaves followed by the stems and the roots. As light levels were decreased from 900 to 675 to 500 μmol/m^2^/s, total flavonoids in leaves of *Labisia pumila* were enhanced by 58, 44 and 29% respectively, compared to 225 μmol/m^2^/s. A similar trend was observed for total phenolics in leaves where at 900 μmol/m^2^/s total phenolics registered the lowest production (2.395 mg gallic acid/g dry weight) compared to 225 μmol/m^2^/s that recorded 5.511 mg gallic acid/g dry weight. The present result was in agreement with the resource allocation hypothesis proposed by Coley *et al.* [46] that hypothesized the production of flavonoids and phenolics would be up-regulated under low light conditions. From correlation Table 2 it was observed that total flavonoids and phenolics have a significant positive correlation with PAL activity (*R*^2^ = 0.818; *P* ≤ 0.05; total flavonoids; *R*^2^ = 0.718; *P* ≤ 0.05; total phenolics), that indicates that up-regulation of production of carbon-based secondary metabolites in the study might be due to an increase in PAL activity under low light conditions [47]. According to Baas [48], the increase in PAL activity might stimulate the production of total flavonoids and phenolics, especially under low light conditions for certain plants. This was supported by a discovery by Wu *et al.* [49], who that found the increase in carbon-based secondary metabolite production for *Photinia fraseri* under low irradiance was due to increase activity of PAL that justifies increased production of these compounds under low light conditions. A similar observation of increased total plant flavonoids and phenolics under low irradiance was also reported by Wand [50] and Liakura *et al.* [51] in their work. It was suggested that increasing phenolic and flavonoid components in shaded plants are related to lower temperatures under low light conditions. High temperature usually increases anthocyanin degradation in grape skin, together with a decrease in the expression of flavonoids biosynthesis [52]. Chan *et al.* [53] reported much greater concentrations of flavones and flavonols in leaves of vegetables that are exposed to shade. This finding is in agreement with Bergquist’s findings [54], which showed that the use of low irradiance is important for the production of baby spinach that are high in flavonoid concentration and composition. According to Coley *et al.* [46], under low light intensity levels, the potential for energy (carbon) acquisition is low, so there will be more available carbon that can be used for the production of plant secondary metabolites. This might explain why total phenolics and flavonoids increased in *L. pumila*.

### 2.2. Anthocyanin Content

Anthocyanin content was found to be influenced by irradiance levels (*P* ≤ 0.01). The accumulation of anthocyanin was found to be the highest under leaves followed by the stem and the lowest in roots. Antocyanin content in leaves of 225, 500 and 665 μmol/m^2^/s was 0.74, 0.57 and 0.39 mg/petunidin g fresh weight, respectively compared to only 0.19 mg/petunidin g fresh weight in 900 μmol/m^2^/s treatments. In the root, anthocyanin content in 900 μmol/m^2^/s treatment plant was 370% lower than the average antocyanin content in the three irradiance levels values (Table 1). The same observation was documented also by Hughes and Smith [55], who found increased anthocyanin content when *Galax urceolata* seedlings were exposed to low light levels. Generally, induction of anthocyanin synthesis varies in relation to light exposure levels [56,57]. Endogenous signals, developmental stage, environmental factors and previous light exposure modify the effect of light on anthocyanin synthesis [58]. The accumulation of antocyanin in the present study under low light might be *Labisia pumila* acclimation under low light to reduce the photoinhibition effect. According to Pirie and Mullins [59], accumulation of antocyanin was one of the plant mechanisms to reduce photoinhibition under low irradiance levels. Anthocyanins are the naturally occurring phenolic compounds responsible for the color of many flowers, fruits, and berries [60]. It is the most important group of water soluble pigments in plants and has beneficial health effects such as antioxidant and anti-inflammatory agents [61]. Anthocyanins are probably the largest group of phenolic compounds in the human diet, and their strong antioxidant activities suggest their importance in maintaining health. Anthocyanin is also important as an antioxidant, which has roles in promoting good health in reducing the risk of chronic disease and also as an anti-inflammatory agent. It was reported by Tamura and Yamagami [62] that anthocyanins possess some positive therapeutic effects, mainly associated with their antioxidant activities.

### 2.3. Net Photosynthesis Rate

In a previous report by Ibrahim and Jaafar [63], *L. pumila* seemed to be a shade-adapted plant. In the study, in the light response curve analysis, the *A*_max_ (maximum photosynthesis) of *L. pumila* increased with a simultaneous decrease in the compensation point and light saturation point when plants were exposed to greenhouse growing conditions with low photosynthetic photon flux density (PPFD), suggesting that these plants were a shade loving species [64]. According to Kitao *et al.* [65] and Patakas *et al.* [66], shade-adapted plants have the ability to increase *A*_max_ and other energy dissipating mechanisms when grown under low light conditions, compared to high light conditions. In the previous study by Jaafar *et al.* [67], *Labisia pumila* leaves were found to be typically thinner, have more surface area, and contain more chlorophyll than those of sun leaves. As a result, *L. pumila* are more efficient at harvesting sunlight at low light levels. The photosynthetic measurement was conducted to characterize the photosynthetic acclimation of *Labisia pumila* under different irradiance levels. It was found that irradiance levels significantly affected the photosynthesis rate. As irradiance levels increase in an ascending order 225 > 500 > 665 > 900 μmol/m^2^/s, the photosynthesis rate decreased steadily (Table 3). The highest photosynthesis rate was obtained when plants were exposed to 225 μmol/m^2^/s (12.71 μmol/m^2^/s) followed by 500 μmol/m^2^/s (9.12 μmol/m^2^/s), 625 μmol/m^2^/s (3.11 μmol/m^2^/s) and the lowest in 900 μmol/m^2^/s (2.11 μmol/m^2^/s). In the current study, photosynthetic rate was found to have a significant positive relationship with production of the secondary metabolites. The same positive relationship between photosynthesis and the production of secondary metabolites was also observed by Ali and Ashraf [68] and Hura *et al.* [69]. The present result also suggests that the production of secondary metabolites in *Labisia pumila* was up-regulated under low irradiance when photosynthetic performance was enhanced.

### 2.4. Maximum Efficiency of Photosystem II (Fv/Fm) and Activity of Photosystem II (Fm/Fo)

The (maximum efficiency of Photosystem II) Fv/Fm and activity of photosystem II (Fm/Fo) was influenced by the irradiance levels (*P* ≤ 0.05). Under high irradiance of 900 μmol/m^2^/s, *Labisia pumila* recorded low Fv/Fm (0.681), followed by irradiance at 675 μmol/m^2^/s (0.711) and 500 μmol/m^2^/s (0.771) and the highest value was documented at 225 μmol/m^2^/s at 0.810 (Table 3). A similar trend was also observed with Fm/Fo where 225 μmol/m^2^/s had registered the highest values of 12.71 compared to 9.12 at 500 μmol/m^2^/s, 3.11 at 675 μmol/m^2^/s and 2.11 at 900 μmol/m^2^/s irradiances. These results imply that photoinhibition may have occurred in *Labisia pumila* plants under high light levels [63] when the values of Fm/Fo decreased with increasing irradiances [70]. In the present study, the decrease in Fv/Fm and Fm/Fo could be attributed to partial deactivation of the PS2 reaction centre under high irradiance [38,71]. As shown in the correlation table, the Fv/Fm and Fm/Fo have significant positive correlations with production of total flavonoids, phenolics, and anthocyanin, suggesting that the production of these compounds was less favored under high irradiance and photoinhibition. The increase in production of secondary metabolites under high Fv/Fm and Fm/Fo was also observed by Chen *et al.* [72] and Spulak [73] on liana and beech plants respectively. In the present study, the increase in Fv/Fm and Fm/Fo values under low light conditions was followed by enhanced net photosynthesis under low light, showing that this plant is a shade-loving plant [74].

### 2.5. Phenyl Alanine Lyase (PAL) Activity

The PAL activity was found to be highest (Table 4) under 225 μmol/m^2^/s (33.71 nM transcinnamic mg^−1^ protein hour^−1^) and the lowest at 900 μmol/m^2^/s that registered 12.32 nM transcinnamic mg^−1^ protein hour^−1^. An increase in the production of flavonoids, phenolics and anthocyanins in the present work could be due to an increase in PAL activities under low irradiance levels. Correlation analysis (Table 2) showed that PAL activity had significant positive relationships with total flavonoids (*R*^2^ = 0.718; *P* ≤ 0.05), phenolics (*R*^2^ = 0.818; *P* ≤ 0.05) and anthocyanins (*R*^2^ = 0.816; *P* ≤ 0.05), which might indicate an up-regulation of plant secondary metabolite production with increased PAL activity. The present result also indicates that under low light levels, the activity of phenylalanine might be increased, which simultaneously enhanced the production of plant secondary metabolites [33,72]. The increase in PAL activity under low light irradiance was also observed by Mohr *et al.* [75] in birch seedlings. These results suggest that up-regulation of production of plant secondary metabolites in *Labisia pumila* under low irradiance could be attributed to the increase in PAL activity. Despite the low light, the imposition of blue and UV-A light also can activate and increase PAL activity in the plant [76–78].

### 2.6. 1,1-Diphenyl-2-picryl-hydrazyl (DPPH) Assay

The purple colored DPPH is a stable free radical, which can be reduced to α,α-diphenyl-β-picryhydrazine (yellow colored) when reacted with antioxidant. The latter interrupts the free radical chain oxidation by donating hydrogen from the hydroxyl group to form a stable end product that does not initiate or propagate further oxidation of lipids [79]. Generally, DPPH antioxidant activity in *Labisia pumila* was found to be the highest on the underside of leaves, followed by the stems and the roots in all levels of irradiances. The DPPH antioxidant activity at 225 μmol/m^2^/s was the highest (62.42–52.21%), followed by the 500 μmol/m^2^/s (51.83–47.73%), 625 μmol/m^2^/s (45.43–40.31%) and the lowest in 900 μmol/m^2^/s (39.21–32.65%), However, DPPH radical scavenging abilities of the extracts of the plant were less than for those of butylated hydroxyl toluene (BHT) (67.81%) and α-tocopherol (78.41%) (Table 5). This study showed that *Labisia pumila* methanolic extract demonstrated inhibiting activity against DPPH free radicals and hence could be used as a radical scavenging agent, acting possibly as the primary antioxidant. This result implies that low irradiance is able to significantly enhance DPPH radical scavenging activity. From the correlation analysis in Table 2, it was shown that total flavonoids, phenolics and anthocyanins had significant positive relationships (*R*^2^ = 0.836; *R*^2^ = 0.826; *R*^2^ = 0.821; *P* ≤ 0.05) respectively with DPPH, implying that high antioxidant power under low irradiation might be contributed by a high content of gallic acid, quercetin and anthocyanin in the plant extract. Previous studies have shown that a combination of polyphenolic compounds plus anthocyanin produced a synergistic effect on DPPH radical scavenging activity [80]. The result from the current study indicated that the high DPPH activity of *Labisia pumila* under low irradiance might be due to high contents of flavonoids, phenolics and anthocyanins in *Labisia pumila* extracts [81].

## 3. Experimental Section

### 3.1. Plant Material and Maintenance

The experiment was carried out in glass houses at Field 10, University Agriculture Park, Faculty of Agriculture Glasshouse Complex, Universiti Putra Malaysia (longitude 101°44′N and latitude 2°58′S, 68 m above sea level) with a mean atmospheric pressure of 1.013 kPa. Three-month old *L. pumila* seedlings of about three months old *Labisia pumila* seedlings of var. *alata* were left for a month to acclimatize in a nursery until ready for the treatments. When the seedlings had reached 4 months of age and they were fertilized with NPK Blue Special at 15 g per plant. The seedlings were planted in a soilless medium containing coco-peat, burnt paddy husk and well composted chicken manure in a 5:5:1 (v/v) ratio in 25 cm diameter polyethylene bags. Day and night temperatures in the greenhouse were maintained at 27–30 °C and 18–21 °C, respectively, and relative humidity from 50 to 60%. All the seedlings were irrigated using overhead mist irrigation, given four times a day or when necessary. Each irrigation session lasted for 7 min. *Labisia pumila* Benth is a shade plant and needs shade for maximum production. The plants were grown under four levels of glasshouse shade (0%, 20%, 40% and 60% shade) using black tildanet. The average light intensity passing through in each shading treatment for (0%, 20%, 40% and 60%) was 900, 625, 500 and 225 μmol/m^2^/s respectively. The photosynthetic photon flux density was measured using LICOR-1412 quantum sensors. During the experiment the photosynthetic photon flux density was in the range of 23–1450 μmol/m^2^/s. The experiment was based on a Randomized Complete Block Design (RCBD) with four replicates. Each treatment consisted of 10 plants, totaling 160 plants in the experiment. Plants were harvested at 16 weeks after planting.

### 3.2. Total Flavonoids and Phenolics Quantification

Extraction and quantification for total phenolics and flavonoids contents followed the method of Jaafar *et al.* [82]. The plant samples were collected early in the morning (8:00–9:00 a.m.) after 16 weeks of treatment. The plant samples were placed in a polyethylene and kept in a refrigerator (5 °C in darkness) for no longer than 12 hours. On completion, the plants were subsequently separated into shoots and roots. The leaves were separated from the stems and oven dried. The stems were cut into small pieces and oven dried at 37 °C to constant weight. After that, an amount of ground tissue samples (0.1 g) was extracted with 80% ethanol (10 mL) in an orbital shaker for 120 minutes at 50 °C. The mixture was subsequently filtered (Whatman™ No.1), and the filtrate was used for the quantification of total phenolics and total flavonoids. Folin–Ciocalteu reagent (diluted 10-fold) was used to determine the total phenolics content of the leaf samples. Two hundred μL of the sample extract was mixed with Follin–Ciocalteau reagent (1.5 mL) and allowed to stand at 22 °C for 5 minutes before adding NaNO_3_ solution (1.5 mL, 60 g·L^−1^). After two hours at 22 °C, absorbance was measured at 725 nm. The results were expressed as mg·g^−1^ gallic acid equivalent (mg·GAE·g^−1^ dry sample). For total flavonoids determination, a sample (1 mL) was mixed with NaNO_3_ (0.3 mL) in a test tube covered with aluminum foil, and left for 5 minutes. Then 10% AlCl_3_ (0.3 mL) was added, followed by the addition of 1 M NaOH (2 mL). Later, the absorbance was measured at 510 nm using a spectrophotometer with rutin as a standard (results expressed as mg·g^−1^ rutin dry sample).

### 3.3 Anthocyanin Content

Anthocyanin content was determined according to Bharti and Khurana [83]. Fresh leaves (1 g) were added in 10 mL acidic methanol (1% v/v HCl) and incubated overnight. Anthocyanin was partitioned from chlorophyll with 10 mL chloroform, followed by adding 9 mL of double deionized water. The test tubes containing the samples were shaken gently and the mixture allowed to settle down. The absorbance was read at 505 nm. Petunidin was used as a standard. Anthocyanin content was recorded as mg/g petunidin fresh weight.

### 3.4. Photosynthesis Rate

The measurement was obtained from a closed infra-red gas analyzer LICOR 6400 Portable Photosynthesis System (IRGA, Licor Inc., Lincoln, NE, USA). The measurements used optimal conditions set of 400 μmol·mol^−1^ CO_2_, 30 °C cuvette temperature, 60% relative humidity with air flow rate set at 500 cm^3^·min^−1^, and modified cuvette conditions of 225, 500, 625 and 900 μmol/m^2^/s respectively, photosynthetically photon flux density (PPFD), according to the irradiance treatment. Gas exchange measurements were carried out between 09:00 to 11:00 a.m., using fully expanded young leaves numbered 3 and 4 from plant apex to record net photosynthesis rate (A). The operation was automatic and the data were stored in the LI-6400 console and analyzed by “Photosyn Assistant” software (version 1.0; Dundee Scientific: Dundee, Scotland, UK, 2000). Several precautions were taken to avoid errors during measurements. Leaf surfaces were cleaned and dried using tissue paper before being enclosed in the leaf cuvette [84].

### 3.5. Chlorophyll Fluorescence

Measurements of chlorophyll fluorescence were taken from a fully expanded leaf of the second leaves. Leaves were darkened for 15 minutes by attaching light-exclusion clips to the central region of the leaf surface. Chlorophyll fluorescence was measured using a portable chlorophyll fluorescence meter (Handy PEA, Hansatech Instruments Ltd., Norwich, UK). Measurements were undertaken at >3000 μmol/m^2^/s and recorded up for 5 seconds [85]. The fluorescence responses were induced by emitting diodes. Measurement of Fo (initial fluorescence), Fm (maximum fluorescence) and Fv (variable fluorescence) were obtained from this procedure. Fv is derived as the differences between Fm and Fo. The mean value of three representative plants was used to represent each sub-plot.

### 3.6. Phenylalanine-Ammonia-Lyase (PAL)

Phenylalanine-ammonia-lyase (PAL) activity was measured using the method described by Martinez and Lafuente [86]. The enzyme activity was determined by measuring spectrophotometrically the production of *trans*-cinnamic acid from L-phenylalanine. Enzyme extract (10 μL) was incubated at 40 °C with 12.1 mM L-phenylalanine (90 μL, Sigma) that were prepared in 50 mM Tris-HCl, (pH 8.5). After 15 minutes of reaction, *trans*-cinnamic acid yield was estimated by measuring increase in the absorbance at 290 nm. A standard curve was prepared by using a *trans*-cinnamic acid standard (Sigma) and the PAL activity was expressed as nM *trans*-cinnamic acid μg^−1^ protein hour^−1^.

### 3.7. DPPH Radical Scavenging Assay

The DPPH free radical scavenging activity of each sample was determined according to the method described by Joyeux *et al.* [87]. A solution of 0.1 mM DPPH in methanol was prepared. The initial absorbance of the DPPH in methanol was measured at 515 nm. An aliquot (40 μL) of an extract was added to 3 mL of methanolic DPPH solution. The change in absorbance at 515 nm was measured after 30 minutes. The antiradical activity (AA) was determined using the following formula:

AA%=100-[(Abs:sample-Abs:empty sample)×100]/Abs:control

The optical density of the samples, the control and the empty samples were measured in comparison with methanol. One synthetic antioxidant, BHT (butylhydroxytoluene) and α-tocopherol were used as positive controls. The antioxidant capacity, based on the DPPH free radical scavenging ability of the extract, was expressed as μmole Trolox equivalent per gram of dried plant material.

### 3.8. Statistical Analysis

Data were analyzed using analysis of variance by SAS version 17 (SAS Institute Inc.: Cary, NC, USA, 2006). Mean separation test between treatments was performed using Duncan multiple range test and standard error of differences between means was calculated with the assumption that data were normally distributed and equally replicated.

## 4. Conclusions

Our results indicate that the manipulation of irradiance levels may be an effective method to increase the expression of secondary metabolite compounds in *Labisia pumila*. Higher total flavonoids, phenolics, and anthocyanin levels were demonstrated in *Labisia pumila* when the irradiance level was at its lowest (225 μmol/m^2^/s). The significant positive correlations of production of total flavonoid, phenolic and anthocyanin contents with Fv/Fm, Fm/Fo and net photosynthesis indicate the occurrence of the up-regulation of production of CBSM under reduced photoinhibition conditions and low irradiance levels. Moreover, at the lowest irradiance level, *Labisia pumila* extracts exhibited significantly higher antioxidant activity (DPPH) than under high irradiance. The high antioxidative effects under low irradiance levels might be due to high availability of total flavonoids, phenolics and anthocyanin in the plant extract. It was also found that the increase in the production of CBSM was attributed to high PAL activity under low irradiance levels, signifying more availability of phenylalanine under these conditions.

## Figures and Tables

**Table 1:** Accumulation and partitioning of total flavonoids and total phenolics in different plant parts of *Labisia pumila* under different irradiance levels.

Irradiance (μmol/m^2^/s)	Plant Parts	Total Flavonoids (mg quercetin/g Dry Weight)	Total Phenolics (mg gallic acid/g Dry Weight)	Anthocyanin (mg/g Fresh Weight)
225	Leaf	2.211 ± 0.013 ^a^	5.511 ± 0.028 ^a^	0.74 ± 0.01 ^a^
	Stem	1.991 ± 0.022 ^a^	4.811 ± 0.029 ^a^	0.67 ± 0.02 ^a^
	Root	1.571 ± 0.013 ^b^	4.571 ± 0.039 ^b^	0.63 ± 0.03 ^a^

500	Leaf	1.547 ± 0.022 ^b^	4.311 ± 0.032 ^b^	0.57 ± 0.12 ^b^
	Stem	1.301 ± 0.030 ^b^	3.971 ± 0.037 ^b^	0.50 ± 0.23 ^b^
	Root	1.241 ± 0.022 ^b^	3.781 ± 0.051 ^c^	0.48 ± 0.12 ^b^

675	Leaf	1.214 ± 0.013 ^c^	3.171 ± 0.021 ^c^	0.39 ± 0.03 ^c^
	Stem	1.021 ± 0.010 ^c^	2.991 ± 0.025 ^c^	0.35 ± 0.03 ^c^
	Root	0.957 ± 0.015 ^c^	2.771 ± 0.040 ^c^	0.30 ± 0.02 ^c^

900	Leaf	0.903 ± 0.025 ^c^	2.395 ± 0.008 ^d^	0.19 ± 0.04 ^d^
	Stem	0.803 ± 0.023 ^d^	1.991 ± 0.011 ^d^	0.15 ± 0.04 ^d^
	Root	0.713 ± 0.026 ^d^	1.711 ± 0.028 ^e^	0.10 ± 0.02 ^d^

All analyses are mean ± standard error of mean (SEM). *N* = 40. Means within a column not sharing a common letter were significantly different at *P ≤* 0.05.

**Table 2:** Correlations among the measured parameters in the experiments.

Parameters	1	2	3	4	5	6	7	8
**1. Flavonoids**	1.000							
**2. Phenolics**	0.917 **	1.000						
**3. Antocyanin**	0.825 *	0.828 *	1.000					
**4. Photosynthesis**	0.829 *	0.826 *	0.817 *	1.000				
**5.** Fv/Fm	0.927 *	0.835 *	0.827 *	0.832 *	1.000			
**6.** Fm/Fo	0.817 *	0.827 *	0.816 *	0.828 *	0.866 *	1.000		
**7. PAL**	0.818 *	0.718 *	0.926 *	0.828 *	0.932 *	0.816 *	1.000	
**8. DPPH**	0.826 *	0.836 *	0.821 *	0.819 *	0.914 *	0.847 *	0.965 *	1.000

Note Fv/Fm = Maximum efficiency of photosystem II; PAL = Phenyl alanine lyase activity and DPPH = 1,1-diphenyl-2-picryl-hydrazyl (DPPH) assay.

* and ** respectively significant at *P* ≤ 0.05 or *P* ≤ 0.01.

**Table 3:** The effect of irradiance levels on photosynthesis, Fv/Fm and Fm/Fo in *Labisia pumila.*

Irradiance (μmol/m^2^/s)	Net Photosynthesis, A (μmol/m^2^/s)	Maximum Efficiency of Photosystem II (Fv/Fm )	Fm/Fo
225	12.71 ± 0.15 ^a^	0.810 ± 0.234 ^a^	12.71 ± 0.01 ^a^
500	9.12 ± 0.45 ^b^	0.771 ± 0.123 ^b^	9.12 ± 0.43 ^b^
675	3.11 ± 0.32 ^c^	0.711 ± 0.213 ^c^	3.11 ± 0.54 ^c^
900	2.11 ± 0.22 ^d^	0.681 ± 0.011 ^d^	2.11 ± 0.44 ^d^

All analyses are mean ± standard error of mean (SEM), *N* = 45. Means within a column not sharing a common single letter were significantly different at *P* ≤ 0.05.

**Table 4:** The effect of irradiance levels on PAL activity in *Labisia pumila.*

Irradiance (μmol/m^2^/s)	PAL Activity (nM transcinnamic mg^−1^ protein hour^−1^)
225	33.71 ± 3.22 ^a^
500	29.82 ± 1.67 ^b^
675	21.71 ± 2.21 ^c^
900	12.32 ± 2.31 ^d^

All analyses are mean ± standard error of mean (SEM), *N* = 40. Means within a column not sharing a common single letter were significantly different at *P* ≤ 0.05.

**Table 5:** DPPH scavenging activities in different parts of three varieties of *Labisia pumila* under different irradiance levels. BHT and α-tocopherol were used as positive controls.

Irradiance (μmol/m^2^/s)	Extract Source	Inhibition % a
225	Leaf	62.42 ± 1.65 ^c^
	Stem	58.14 ± 1.09 ^c^
	Root	52.21 ± 1.08 ^c^

500	Leaf	51.83 ± 1.05 ^d^
	Stem	49.11 ± 0.98 ^d^
	Root	47.73 ± 0.43 ^d^

675	Leaf	45.43 ± 0.23 ^e^
	Stem	44.74 ± 0.98 ^e^
	Root	40.31 ± 1.21 ^e^

900	Leaf	39.21 ± 2.22 ^f^
	Stem	37.16 ± 1.21 ^f^
	Root	32.65 ± 3.21 ^f^

**Controls**	BHT	67.81 ± 1.34 ^b^

	α-tocopherol	78.41 ± 1.24 a

All analyses are mean ± standard error of mean (SEM), *N* = 45. Means within a column not sharing a common single letter were significantly different at *P* ≤ 0.05.

a Results expressed in percent of free radical inhibition.

## References

[1] Cai Y.Z., Sun M., Xing J., Luo Q., Corke H. (2006). Structure-radical scavenging activity relationships of phenolic compounds from traditional Chinese medical plants. Life Sci.

[2] Contini M., Baccelloni S., Massantini R., Anelli G. (2008). Extraction of natural antioxidants from hazelnut (*Corylus avellana* L.) shell and skin wastes by long maceration at room temperature. Food Chem.

[3] Huang M.T., Ho C.T., Lee C.Y. (1992). Phenolic Compounds in Food and Their Effects on Health II: Antioxidants and Cancer Prevention. American Chemical Society Symposium Series 507.

[4] Hertog M.G.L., Fesrens E.J.M., Hollman P.C.H., Katan M.B., Kromhout D. (1993). Dietary antioxidant flavonoids and risk of coronary heart disease: The Zutphen elderly study. Lancet.

[5] Diaz M.N., Frei B., Vita J.A., Keaney J.F. (1997). Antioxidants and atherosclerotic heart disease. N. Eng. J. Med.

[6] Ito N., Hirose M. (1989). Antioxidants-carcinogenic and chemo-preventive properties. Adv. Cancer Res.

[7] Ness A.R., Powles J.W. (1997). Fruit and vegetables and cardiovascular disease: A review. Int. J. Epidemiol.

[8] Cowan M.M. (1999). Plant products as antimicrobial agents. Clin. Microbiol. Rev.

[9] Shetty K. (1997). Biotechnology to harness the benefits of dietary phenolics: Focus on Lamiaceae. Asia Pac. J. Clin. Nutr.

[10] Fine P.V.A., Miller Z.J., Mesones I., Irazuzta S., Appel H.M., Stevens M.H.H. (2006). The growth defense trade off and habitat specialization by plants in Amazonian forest. Ecology.

[11] Briskin D.P., Gawienowski M.C. (2001). Differential effects of light and nitrogen on production of hypericins and leaf glands in *Hypericum perforatum*. Plant Physiol.

[12] Kurata H., Matsumura S., Furusaki S. (1997). Light irradiation causes physiological and metabolic changes for purine alkaloid production by a Coffea Arabica cell suspension culture. Plant Sci.

[13] Hemm M.R., Rider S.D., Ogas J., Murry D.J., Chapple C. (2004). Light induces phenylpropanoids metabolism in *Arabidopsis* roots. Plant J.

[14] Jaakola L., Maatta-Riihinen K., Karenlampi S., Hohtola A. (2004). Activation of flavonoid biosynthesis by solar radiation in bilberry (*Vaccinium myrtillus* L.) leaves. Planta.

[15] Bernards M.A., Ellis B.E. (1991). Phenylalanine ammonia-lyase from tomato cell cultures inoculated with *Verticillium alboatrum*. Plant Physiol.

[16] Kovacik J., Backor M. (2007). Phenylalanine ammonia-lyase and phenolic compounds in Chamomile tolerance to cadmium and copper excess. Water Air Soil Pollut.

[17] Jansen M.A.K., Hectors K., O’Brien N.M., Guisez Y., Potters G. (2008). Plant stress and human health: Do human consumers benefit from UV-B acclimated crops?. Plant Sci.

[18] Fritz C., Palacios N., Fiel R., Stitt M. (2006). Regulation of secondary metabolism by the carbon-nitrogen status in tobacco: Nitrate inhibits large sectors of phenylpropanoids metabolism. Plant J.

[19] Coelho G.C., Rachwal M.F.G., Dedecek R.A., Curcio G.R., Nietsche K., Schenkel E.P. (2007). Effect of light intensity on methylxanthine contents of *Ilex paraguariensis* A. St. Hil. Biochem. Syst. Ecol.

[20] Ralphs M.H., Manners G.D., Gardner D.R. (1998). Influence of light and photosynthesis on alkaloid concentration in larkspur. J. Chem. Ecol.

[21] Zhan J.C., Wang L.J., Huang W.D. (2002). Effects of low light environment on the growth and photosynthetic characteristics of grape leaves. J. China Agric.

[22] Paez A., Gebre G.M., Gonzalez M.E., Tschaplinski T.J. (2000). Growth, soluble carbohydrates, and aloin concentration of Aloe vera plants exposed to three irradiance levels. Environ. Exp. Bot.

[23] Chauser-Volfson E., Gutterman Y. (1998). Content and distribution of anthrone C-glycosides in the South African arid plant species Aloe mutabilis growing in the direct sunlight and the shade in the Negev Desert of Israel. J. Arid Environ.

[24] Wang Y., Gao L., Wang Z., Liu Y., Sun M., Yang D., Wei C., Xia T. (2012). Light induced expression of genes involved in phenylpropanoid biosynthesis pathway in callus of tea. Sci. Hort.

[25] Peng Y., Bo Y. (2012). Extraction mechanism of total flavonoid on red kidney bean by microwave and light wave radiation. Adv. Mater. Res.

[26] Brechner M.L., Albright L.D., Westa L.A. (2011). Effect of UVB on secondary metabolites of St John’s Wort (*Hypericum perforatum* L.) grown in controlled environment. Photochem. Photobiol.

[27] Chazdon R.L., Pearcy R.W., Lee D.W., Fetcher N., Mulkey S.S., Chazdon R.L., Smith A.P. (2003). Photosynthetic Response of Tropical Forest Plants to Contrasting Light Environments. Tropical Forests Plant Ecophysiology.

[28] Demmig-Adams B., Adams W.W. (1992). Carotenoid composition in sun and shade leaves of plants with different life forms. Plant Cell Environ.

[29] Long S.P., Humphries S., Falkowski P.G. (1994). Photoinhibition of photosynthesis in nature. Annu. Rev. Plant Physiol. Plant Mol. Biol.

[30] Krause G.H., Koroleva O.Y., Dalling W., Winter K. (2001). Acclimation of tropical tree seedlings to excessive light in simulated tree-fall gaps. Plant Cell Environ.

[31] Laisk A., Eichelmann H., Oja V., Rasulov B., Padu E., Bichele I., Pettai H., Kull O. (2005). Adjustment of leaf photosynthesis to shade in a natural canopy: Rate parameters. Plant Cell Environ.

[32] Martinez C.A., Maestri M., Mathis P. (1995). Photoinhibition of Photosynthesis in Andean Potato (*Solanum* spp.) Species Differing in Frost Resistance. Photosynthesis: From Light to Biosphere.

[33] Kitao M., Yoneda R., Tobita H., Matsumoto Y., Maruyama Y., Arifin A., Asan A., Muhamad M. (2006). Susceptibility to photoinhibition in seedlings of six tropical fruit tree species native to Malaysia following transplantation to a degraded land. Trees.

[34] Close D., McArthur C., Paterson S., Fitzgerald H., Walsh A., Kincade T. (2003). Photoinhibition: A link between effects of the environment on eucalypt leaf chemistry and herbivory. Ecology.

[35] Jaafar H.Z.E., Mohamed H.N.B., Rahmat A. (2008). Accumulation and partitioning of total phenols in two varieties of *Labisia pumila* Benth. under manipulation of greenhouse irradiance. Acta Hort.

[36] Stone B.C. (1988). Notes on the genus *Labisia Lindyl* (Myrsinaceae). Malay. Nat. J.

[37] Rozihawati Z., Aminah H., Lokman N. (2003). Preliminary trials on the rooting ability of *Labisia pumila* cuttings.

[38] Ibrahim M.H., Jaafar H.Z.E., Rahmat A., Rahman Z.A. (2010). The Relationship between phenolics and flavonoids production with total non structural carbohydrate and photosynthetic rate in *Labisia pumila* Benth. under high CO_2_ and nitrogen fertilization. Molecules.

[39] Ibrahim M.H., Jaafar H.Z.E. (2011). Enhancement of leaf gas exchange and primary metabolites, up-regulate the production of secondary metabolites of *Labisia pumila* blume seedlings under carbon dioxide enrichment. Molecules.

[40] Ibrahim M.H., Jaafar H.Z.E. (2011). The influence of carbohydrate, protein and phenylanine ammonia lyase on up-regulation of production of secondary metabolites (total phenolics and flavonoid) in *Labisia pumila* (Blume) Fern-Vill (Kacip Fatimah) under high CO_2_ and different nitrogen levels. Molecules.

[41] Ibrahim M.H., Jaafar H.Z.E. (2011). The relationship of nitrogen and C/N on secondary metabolites and antioxidant activities in three varieties of Malaysia kacip Fatimah (*Labisia pumila* Blume). Molecules.

[42] Ibrahim M.H., Hawa Z.E.J. (2011). Carbon dioxide fertilization enhanced antioxidant compounds in Malaysian kacip Fatimah (*Labisia pumila* Blume). Molecules.

[43] Norhaiza M., Maziah M., Hakiman M. (2009). Antioxidative properties of leaf extracts of popular Malaysian herb, *Labisia pumila*. J. Med. Plant Res.

[44] Karimi E., Jaafar H.Z.E., Ahmad S. (2011). Phytochemical analysis and antimicrobial activites of methanolics extracts of leaf, stem and root from different varieties of *Labisia pumila* Benth. Molecules.

[45] Jamia A.J., Ibrahim J., Khairana H., Juriyati H. (2004). Perkembangan Penyelidikan dan Pembangunan Kacip Fatimah.

[46] Coley P.D., Bryant J.P., Chapin F.S. (1985). Resource availability and plant antiherbivore defense. Science.

[47] Guo R., Yuan G., Wang Q. (2011). Effect of sucrose and mannitol on the accumulation of health-promoting compounds and the activity of metabolic enzymes in broccoli sprouts. Sci. Hort.

[48] Baas W.J., Lambers H., Cambridge M., Konings H., Pons T.L. (1989). Secondary Plant Compounds, Their Ecological Significance and Consequences for the Carbon Budget. Introduction of the Carbon/Nutrient Cycle Theory. Causes and Consequences of Variation in Growth Rate and Productivity of Higher Plant.

[49] Wu L., Gao Y., Huang W., Saig H. (2011). Effects of EU3^+^ on anthocyanin content and PAL activity in potted *Photinia fraseri* under different light intensity. Chin. Rare Earth Soc.

[50] Wand S.J.E. (1995). Concentration of ultraviolet-B radiation absorbing compounds in leaves of a range of fynbos species. Vegetatio.

[51] Liakoura V., Bornman J.F., Karabourniotis G. (2003). The ability of abaxial and adaxial epidermis of sun and shade leaves to attenuate UV-A and UV-B radiation in relation to the UV absorbing capacity of the whole leaf methanolic extract. Physiol. Plant.

[52] Mori K., Goto-Yamamoto N., Kitayama M., Hashizume K. (2007). Loss of anthocyanins in red-wine grape under high temperature. Exp. Bot.

[53] Chan E.W.C., Lim Y.Y., Wong F.L., Lianto F.S. (2008). Antioxidant and tyrosinase inhibition properties of leaves and rhizomes in ginger species. Food Chem.

[54] Bergquist S., Gertsson U., Nordmark L.Y., Olsson M.E. (2007). Effects of shade nettings, sowing time and storage on baby spinach flavonoids. J. Sci. Food Agric.

[55] Hughes N.M., Smith W.K. (2007). Attenuation of incident light in *Galax urceolata* (Diapensiaceae): Concerted influence of adaxial and abaxial anthocyanic layers on photoprotection. Am. J. Bot.

[56] Gould K.S., Dudle D.A., Neufeld H.S. (2010). Why some stems are red: Cauline anthocyanins shield photosystem II against high light stress. J. Exp. Bot.

[57] Mancinelli A.L., Shropshire W., Mohr H. (1983). The Photoregulation of Anthocyanin Synthesis. Photomorphogenesis.

[58] Lee D.W., Lowry J.B., Stone B.C. (1979). Abaxial anthocyanin layer in leaves of tropical rain forest plants: Enhancer of light capture in deep shade. Biotropica.

[59] Pirie A., Mullins M.G. (1976). Changes in anthocyanin and phenolics content of grapevine leaf and fruit tissues treated with sucrose, nitrate, and abscisic acid. Plant Physiol.

[60] Chalker-Scott L. (1999). Environmental significance of anthocyanins in plant stress responses. Photochem. Photobiol.

[61] Wang H., Nair M.G., Strasburg G.M., Chang Y.C., Booren A.M., Gray J.I., DeWitt D.L. (1999). Antioxidative activity of monoacylated anthocyanins isolated from Muscat bailey A grape. J. Nat. Prod.

[62] Tamura H., Yamagami A. (1994). Antioxidative activity of monoacylated anthocyanins isolated from Muscat bailey A grape. J. Agric. Food Chem.

[63] Ibrahim M.H., Jaafar H.Z.E. (2011). Photosynthetic capacity, photochemical efficiency and chlorophyll content of three varieties of *Labisia pumila* Benth. exposed to open field and greenhouse growing conditions. Acta Physiol. Plant.

[64] Lambers H., Chapin F.S., Pons T.L. (1998). Plant Physiological Ecology.

[65] Kitao M., Lei T.T., Koike T., Tobita H., Maruyama Y. (2000). Susceptibility to photoinhibition of three deciduous broadleaf tree species with different successional traits raised under various light regimes. Plant Cell Environ.

[66] Patakas A., Kofidis G., Bosabalis A.M. (2003). The relationship between CO_2_ transfer mesophyll resistance and photosynthetic efficiency in grapevine cultivars. Sci. Hortic.

[67] Jaafar H.Z.E., Ibrahim M.H., Philip E. (2009). Leaf gas exchange properties of three varieties of *Labisia pumila* Benth. under greenhouse conditions. J. Trop. Plant Physiol.

[68] Ali Q., Ashraf M. (2011). Induction of drought tolerance in maize (*Zea mays* L.) due to exogenous application of trehalose: Growth, photosynthesis, water relations and oxidative defence mechanism. J. Agric. Crop Sci.

[69] Hura T., Hura K., Grzesiak M. (2011). Soil drought applied during the vegetative growth of triticale modifies the physiological and biochemical adaptation to drought during the generative development. J. Agric. Crop Sci.

[70] Govindjee A.J., Downton W.J.S., Fork D.C., Armond P.A. (1981). Chlorophyll a fluorescence transient as an indicator of water potential of leaves. Plant Sci. Lett.

[71] Lovelock C.E., Jebb M., Osmond C.B. (1994). Photoinhibition and recovery in tropicalplant species: Response to disturbance. Oecologia.

[72] Chen Y.J., Zhu S.D., Cao K.F. (2008). Comparison of the eco-physiological characteristics between seedlings of lianas and trees under two light irridiances. Acta Ecol. Sin.

[73] Špulák O. (2008). Assimilation apparatus variability of beech transplants grown in variable light conditions of blue spruce shelter. J. For. Sci.

[74] Schiefthaler U., Russel W., Bolhar-Nordenkampf H.R., Crithely C. (1997). Photoregulation and photodamage in *Schefflera arboricola* leaves adapted to different light environment. Aust. J. Plant Physiol.

[75] Mohr H., Drumm H., Schmidt R., Steinitz B. (1979). The effect of light pretreatments on phytochrome-mediated induction of anthocyanin and of phenylalanine ammonia-lyase. Planta.

[76] Christie J.M., Jenkins G.I. (1996). Distinct UV-B and UV-A/blue light signal transduction pathways induce chalcone synthase gene expression in *Arabidopsis* cells. Plant Cell.

[77] Fuglevand G., Jackson J.A., Jenkins G.I. (1996). UV-B, UV-A, and blue light signal transduction pathways interact synergistically to regulate chalcone synthase gene expression in *Arabidopsis*. Plant Cell.

[78] Long J.C., Jenkins G.I. (1998). Involvement of plasma membrane redox activity and calcium homeostasis in the UV-B and UV-A/blue light induction of gene expression in *Arabidopsis*. Plant Cell.

[79] Sherwin E.R. (1978). Oxidation and antioxidants in fat and oil processing. J. Am. Oil Soc.

[80] Lavola A., Julkunen-Tiitto R., de la Rosa T.M., Lehto T., Aphalo P.J. (2000). Allocation of carbon to growth and secondary metabolites in birch seedlings under UV-B radiation and CO_2_ exposure. Physiol. Plantarum.

[81] Murakami M., Yamaguchi T., Takamura H., Matoba T. (2003). Effects of ascorbic acid and tocopherol on antioxidant activity of polyphenolic compounds. Food Chem. Toxicol.

[82] Jaafar H.Z.E., Ibrahim M.H., Por L.S. (2010). Effects of CO_2_ Enrichment on Accumulation of Total Phenols, Flavonoid and Chlorophyll Content in Two Varieties of *Labisia pumila* Benth. exposed to Different Shade Levels.

[83] Bharti A.K., Khurana J.P. (2003). Molecular characterization of transparent testa (tt) mutants of *Arabidopsis thaliana* (ecotype Estland) impaired in flavonoid biosynthesic pathway. Plant Sci.

[84] Ibrahim M.H., Jaafar H.Z.E., Rahmat A., Zaharah A.R. (2011). Effects of nitrogen fertilization on synthesis of primary and secondary metabolites in three varieties of kacip Fatimah (*Labisia pumila* Blume). Int. J. Mol. Sci.

[85] Ibrahim M.H., Jaafar H.Z.E., Haniff M., Yusop R. (2010). Changes in the growth and photosynthetic patterns of oil palm (*Elaeis guineensis* Jacq.) seedlings exposed to short term CO_2_ enrichment in a closed top chamber. Acta Physiol. Plant.

[86] Martinez-Tellez M.A., Lafuente M.T. (1997). Effects of high temperature conditioning on ethylene, phenylalanine ammonia lyase, peroxidase and polyphenol oxidase in flavedo of chilled “Fortune” mandarin fruit. J. Plant Physiol.

[87] Joyeux M., Lobstein A., Mortier F. (1995). Comparative antilipoperoxidant, antinecrotic and scavenging properties of terpenes and biflavones from Gingko and some flavonoids. Planta Med.

